# Successes and final challenges along the HIV care continuum with transwomen in San Francisco

**DOI:** 10.1002/jia2.25270

**Published:** 2019-04-29

**Authors:** Glenda N Baguso, Caitlin M Turner, Glenn‐Milo Santos, H Fisher Raymond, Carol Dawson‐Rose, Jess Lin, Erin C Wilson

**Affiliations:** ^1^ Department of Community Health Systems University of California San Francisco San Francisco CA USA; ^2^ San Francisco Department of Public Health Center for Public Health Research San Francisco USA

**Keywords:** transgender women, HIV, HIV care continuum, HIV continuum, viral load, engagement to care

## Abstract

**Introduction:**

To examine the HIV care continuum for transwomen living in San Francisco and to determine factors associated with poor HIV‐related health outcomes.

**Methods:**

Data were collected from 2016 to 2017 with transwomen in San Francisco. Respondent‐driven sampling (RDS) was used to recruit a population‐based sample. Bivariate associations were assessed, and RDS‐weighted multivariable logistic regression was used to identify associations between exposures and outcomes along the HIV care continuum.

**Results:**

Of the 123 self‐identified transwomen in this analysis, ages ranged from 23 to 71 years with a majority identifying as Latina (40.8%) and African American (29.2%). An estimate of 14.3% of participants were not engaged in care, 13% were not currently on antiretroviral therapy (ART), 22.2% had a self‐reported detectable viral load and 13.5% had unknown viral load. Those using hormones had lower odds of *not* being on ART compared to those who did not use hormones. Those with unstable housing had a higher relative risk ratio of having a detectable viral load. Those who experienced both anti‐trans discrimination and racism had higher odds of *not* being in HIV care.

**Conclusions:**

San Francisco has made substantial progress engaging transwomen in the HIV care continuum, but the final push to ensure viral suppression will require addressing social determinants. Future interventions to increase HIV care engagement, ART use and viral suppression among transwomen must address housing needs and risks related to the overlapping effect of both anti‐trans discrimination and racism.

## 1 Introduction

Incident cases of HIV in the United States declined 18% over the period of 2008 to 2014 except in key populations such as transgender women (transwomen) 1. The estimated HIV prevalence among transwomen in the US is 21.7% 2, higher than among almost all key populations at elevated risk of HIV. Prioritizing transwomen in the response to reducing HIV is important to ending the AIDS epidemic 3. An important pillar in the response is positive HIV care continuum indictors of effective diagnosis, treatment access and viral suppression 3.

Studies that assess transwomen's HIV care continuum indicators, specifically access to HIV care, antiretroviral therapy (ART) utilization and viral suppression are limited. The few studies we found indicated that transwomen have low HIV testing rates and low awareness of HIV status, low engagement and retention in HIV care, low medication adherence and low virologic suppression rates 4, 5, 6. Suboptimal HIV engagement rates were found among transwomen from Brazil and US transgender youth 7, 8. African American transwomen were found to have even poorer reported viral suppression (24.5%) 9. Past data from transwomen in San Francisco 2014 found an HIV prevalence of 39%, 87% accessed care, 65% were on ART and 44% were viral suppressed. 10.

In 2013, a multisector effort in San Francisco was initiated to make a concerted push towards eliminating HIV in the city 11. A study measuring changes along the HIV care continuum in San Francisco found significant improvements in time to ART initiation and viral suppression after diagnosis and more time from HIV to an AIDS diagnosis. However, data were not disaggregated by gender group 11. This study was conducted to characterize HIV care continuum indicators among transwomen in San Francisco in 2016 after initiation of efforts to eliminate HIV, and to identify factors associated with HIV care access, ART use and viral suppression. Results from this study can be used to inform policies and programmes to improve HIV care outcomes among this highly affected key population.

## 2 Methods

### 2.1 Study sample and recruitment process

We conducted a secondary analysis of HIV behavioural surveillance data collected from June 2016 to March 2017 from the Transwomen Empowered to Advance Community Health 3 (TEACH 3) study in San Francisco. Eligible participants were 18 years of age or older, lived in San Francisco and self‐identified as a gender different from that typically associated with a male sex assigned at birth (e.g. man). Respondent‐driven sampling (RDS) was used to recruit self‐identified adult transwomen residing in San Francisco. The initial 15 participants or “seeds” came from diverse backgrounds by race/ethnicity, education and HIV status. Seeds were asked to recruit up to 10 participants from their social networks who met eligibility criteria for the study. This recruitment process continued until equilibrium was reached with 318 transwomen (mean recruitment chain = 6; range 1 to 15). For every successful recruit enrolled into the study, the participant recruiter received $20.00 up to a maximum of 10 recruits. After participants provided consent for study procedures, interviewers administered surveys with a handheld computer. All participants received $65.00 for completing the survey, HIV and HCV testing. The Committee on Human Research at the University of California San Francisco approved the study. This analysis focused on the subset of 123 transwomen (38.7%) who laboratory‐tested reactive for HIV antibodies in the study.

### 2.2 Measurements

#### 2.2.1 HIV testing

Regardless of self‐reported HIV status, an INSTI^®^ HIV‐1/HIV‐2 Rapid Antibody Test was offered to all participants. Positive rapid antibody test results for those who did not know their status were confirmed using Determine™ HIV‐1/2 Ag/AB Rapid test. For those who self‐reported and were confirmed HIV positive with the rapid antibody test, no second test was conducted.

#### 2.2.2 Sociodemographic factors

The following demographic factors and social characteristics were used for this analysis: age (20 to 29, 30 to 39, 40 to 49, 50 to 59 and 60 to 75 years), gender identity (female, transgender female, androgynous, genderqueer, questioning or additional gender), race (Asian, African American, Native American, Native Hawaiian or Pacific Islander, White, Latina or Other), education (never attended school, grades 1 to 8, grades 9 to 11, completed high school, completed GED, AA degree, Technical degree, some college, Bachelor's degree or any postgraduate studies), born in the US (yes/no), housing status (unstable/stable), living as fulltime as their self‐reported gender identity (yes/no) and current hormone use (yes/no). In order to analyse the small number of participants who identify as androgynous, genderqueer, questioning or additional gender (n = 6), we included these participants under the umbrella term of “transgender.” Transgender women were compared to those who identified as “female.” Education was re‐coded as less than a high school degree, high school or equivalent degree, and more than high school education.

We identified descriptive population estimates of transwomen and the HIV care continuum based on race. No participants in this analysis identified as Native American. Due to small participant numbers, Native Hawaiian, Pacific Islander, Asian, White and participants who identified as multiple races were included in bivariate and multivariable analysis as Other.

#### 2.2.3 Alcohol and substance use

Consistent with the National HIV Behavioral Surveillance survey 12, the TEACH 3 survey assessed alcohol and substance use. For this analysis, substance use in the past 12 months (e.g. “Did you use any substances in the past 12 months?”) was a dichotomous variable (yes/no). Alcohol use (e.g. “On a typical day when you drank alcohol in the past 12 months, how many drinks did you have?”) was dichotomized based on criteria from the National Institute on Alcohol Abuse and Alcoholism 13: one episode of heavy drinking (>4 drinks in one sitting) was considered high‐risk for alcohol abuse and other disorders, while four drinks or less was considered low‐risk.

#### 2.2.4 Experiences of Discrimination Scale

The Experiences of Discrimination Scale (EOD) was used to measure anti‐trans discrimination and racism in the following settings: school, hiring, work, getting housing, medical care, store, financial, public and police 14. The EOD scale was modified with an additional question regarding discrimination “while staying in a shelter, SRO, or residential treatment.” For each of the aforementioned settings, the 10‐item EOD asked (yes/no) “Have you ever experienced discrimination, been prevented from doing something, or been hassled or made to feel inferior because of your gender identity or presentation, or race ethnicity or color?” Each question verifying setting‐based discrimination was followed by a question clarifying if the experienced discrimination was related to gender identity or presentation, race/ethnicity or both. Situation scores for gender identity, race and both gender identity and race were skewed and therefore dichotomized (yes/no) as in past studies 15. Cronbach's alpha was 0.76 for the present study.

#### 2.2.5 Outcome variables

We conducted bivariate analysis using RDS‐weighted logistic regression models to evaluate associations between demographics, substance use, discrimination and HIV care engagement and ART adherence and detectable or unknown viral loads. HIV care continuum outcome variables for this study assessed whether participants accessed HIV care, were currently on ART and had a detectable or unknown viral load. HIV care access and ART use were self‐reported and assessed as binary variables (yes/no). Viral load was self‐reported and categorized as undetectable, detectable or unknown. Outcomes for logistic regressions were coded in such a way that those currently in care and currently on ART were the reference group. Undetectable participants were the reference group for the viral load outcome (≤50 copies/mL). The focus on those not in HIV care, not on ART and with a detectable or unknown viral load presents a profile of transwomen most in need of interventions.

### 2.3 Statistical analyses

The use of RDS in analyses lies in the ability to generate population estimates from the sample using various weights based on certain assumptions 16, 17. In order to accurately estimate a hidden population with a large sample fraction, Gile's Successive Sampling (SS) weights were assigned using the RDS Analyst 16, 17. Our study has a high sample fraction of one‐third of transwomen with lower SES 10, 18, 19. RDS‐weighted logistic regressions were conducted in Stata 15 (StataCorp. 2017, College Station, TX).

Descriptive statistics are provided that summarize the demographic data on transwomen living with HIV in this sample. Due to the paucity of research on the HIV care continuum among transwomen and the exploratory nature of this study, we analysed outcomes independently rather than conditional upon each other. For the outcomes of in care and on ART, analysis was conducted with binary endpoints of yes/no. Odds ratio was used to compare these two endpoints. Whereas multinomial analysis was conducted with viral load due to the three endpoints of undetectable, detectable or unknown viral load. Relative risk ratios were conducted to compare the three probabilities. Furthermore, our multivariable analyses used a model‐building algorithm to select covariates with *p *<* *0.25 in bivariate analysis 20. All *p* values are two‐sided. Population estimates are reported.

## 3 Results

Of the 123 transwomen who laboratory‐tested reactive for HIV, 118 participants self‐reported HIV‐positive status that laboratory tests confirmed and 5 (4.1%) transwomen self‐reported a negative or unknown HIV status but laboratory‐tested positive for HIV. There were six participants who identified as androgynous, genderqueer, questioning or additional gender. These participants were analysed under the term “transgender.” Demographics based on population estimates are summarized in Table 1. Population estimates of HIV care engagement and ART use based on race/ethnicity were lower for transwomen of colour (African American: 82.5% HIV care, 79.1% on ART; Hispanic/Latina: 82.2% HIV care, 90.9% on ART; Multiple Races: 91.6% HIV care, 87.0% on ART) than Whites (100% HIV care, 100% on ART), with Asians having the lowest HIV care engagement (74.7%), ART use (74.7%) and undetectable viral load (54.3%). Rates were lowest across all races for reported viral suppression (African American: 69.3%; Hispanic/Latina: 68.4%; White: 61.6%; Multiple Race: 54.4%). These estimates did not adjust for housing or discrimination and is solely based on race. Of transwomen living with HIV, the population estimate of those not engaged in HIV care was 14.3%, 13% for those not currently on ART, 22.2% with a self‐reported detectable viral load and 13.5% with an unknown viral load.

**Table 1:** Socio‐demographics with RDS‐weighted estimates for transwomen living with HIV in San Francisco, 2016 to 2017 (n = 123)

Demographics	Sample % (count)	Population estimates% (95% CI)^a^
Age		
20 to 29 years (reference)	8.94 (11)	9.87 (4.13 to 15.61)
30 to 39 years	21.95 (27)	24.73 (13.41 to 36.12)
40 to 49 years	27.64 (34)	24.42 (15.13 to 33.57)
50 to 59 years	35.59 (45)	37.78 (24.79 to 50.81)
60 to 71 years	4.88 (6)	3.21 (1.05 to 5.39)
Gender‐female identity		
Transwoman	66.67 (82)	65.23 (54.09 to 76.28)
Female	33.33 (41)	34.77 (23.72 to 45.91)
Race/ethnicity		
Other	27.64 (34)	29.98 (18.81 to 41.18)
African American	34.96 (43)	29.22 (18.26 to 40.28)
Latina	37.40 (46)	40.80 (27.57 to 53.89)
Education		
Less than high school	34.15 (42)	35.92 (23.95 to 47.68)
High school diploma	36.59 (45)	40.21 (29.13 to 51.50)
More than high school	29.27 (36)	23.87 (15.38 to 32.35)
Born in the US (yes)	72.36 (89)	72.99 (61.39 to 84.55)
No	27.64 (34)	27.01 (20.53 to 38.41)
Unstable housing (yes)	64.23 (79)	70.31 (61.59 to 79.47)
No	35.77 (44)	29.69 (20.53 to 38.41)
Full‐time female (yes)	32.52 (40)	33.08 (22.18 to 44.16)
No	67.48 (83)	66.92 (55.84 to 77.82)
Hormone use (yes)	70.73 (87)	64.58 (53.55 to 75.47)
No	29.27 (36)	35.42 (24.53 to 46.45)
Alcohol use in past 12 months (yes; >4 drinks in a sitting)	8.94 (11)	4.58 (2.44 to 6.71)
No	91.06 (112)	95.42 (93.29 to 97.56)
Substance use in past 12 months (yes)	54.47 (67)	51.57 (39.41 to 63.48)
No	45.53 (56)	48.43 (36.52 to 60.59)
Gender discrimination (yes)	72.36 (89)	66.85 (54.63 to 79.08)
No	27.64 (34)	33.15 (20.92 to 45.37)
Race discrimination (yes)	18.70 (23)	14.88 (7.61 to 22.09)
No	81.30 (100)	85.12 (77.91 to 92.38)
Gender and race discrimination (yes)	71.54 (88)	72.66 (62.34 to 82.89)
No	28.46 (35)	27.34 (17.11 to 37.66)

RDS, respondent‐driven sampling.

a Confidence Intervals computed using the Gile's bootstrap method from RDS Analyst.

In order to characterize discrimination across race, we added a Table 2. We found that African American transwomen still experience more racism and a combination of racism and anti‐trans sentiment than other race/ethnicity groups.

**Table 2:** Anti‐trans, racism and both discrimination based on race in San Francisco, 2016 to 2017 (n = 123)

Race	Anti‐trans, %	Racism, %	Both, %
Other	29.21	13.04	19.32
African American	35.58	47.83	40.91
Latina	38.20	39.13	39.77

### 3.1 Correlates of not accessing HIV care

The population estimate of transwomen not engaged in HIV care was 14.3%. Bivariate analysis and multivariable analysis with RDS‐weighted prevalence of those who were not engaged in HIV care are shown in Table 3. In bivariate analyses, transwomen who reported both anti‐trans discrimination and racism in one or more setting had 6.28 times the odds of not being engaged in HIV care than those who did not report both anti‐trans discrimination and racism (95% CI: 1.37 to 28.70; *p *=* *0.018). Age, education, substance use, racism only and both anti‐trans discrimination and racism were added to the final model for those who were not engaged in HIV care. In the multivariable logistic regression model, transwomen who were 40 to 49 years had significantly lower adjusted odds ratio (aOR) of not being in HIV care compared to the 20 to 29 years old group (aOR: 0.08; 95% CI: 0.01 to 0.74; *p *=* *0.026). Those who experienced anti‐trans discrimination and racism together had 5.14 times higher odds of not being in care (95% CI: 1.07 to 24.68; *p *=* *0.041). No other significant demographic or psychosocial associations were found.

**Table 3:** Bivariate and multivariable analysis: RDS‐weighted estimates of transwomen living with HIV not in HIV care in San Francisco, 2016 to 2017 (n = 123)

Demographics	Not in HIV care Reference group = in care	
	OR (95% CI), *p*	Adjusted odds ratio (95% CI), *p*
Age (23 to 71 years)		
20 to 29 years (reference)	*‐*	‐
30 to 39 years	*1.56 (0.20 to 12.13), p = 0.668*	0.53 (0.06 to 4.72), *p *=* *0.571
40 to 49 years	*0.20 (0.03 to 1.20), p = 0.078*	**0.08 (0.01 to 0.74), ** ***p *** **=** *** *** **0.026**
50 to 59 years	*0.35 (0.05 to 2.37), p = 0.278*	0.20 (0.02 to 2.00), *p *=* *0.166
60 to 71 years	*0.52 (0.04 to 7.04), p = 0.621*	0.36 (0.02 to 6.93), *p *=* *0.492
Gender identity		
Female (transgender women reference)	1.02 (0.22 to 4.62), *p *=* *0.981	a
Race/ethnicity		
Other (reference)	‐	a
African American	3.06 (0.51 to 18.21), *p *=* *0.217	
Latina	3.12 (0.40 to 24.54), *p *=* *0.278	
Education		
Less than high school (reference)	*‐*	‐
High school diploma	*0.15 (0.02‐ 1.05), p = 0.056*	0.12 (0.01 to 1.10), *p *=* *0.061
More than high school	*0.94 (0.17‐ 5.32), p = 0.942*	0.95 (0.18 to 5.04), *p *=* *0.947
Born in the US		
Yes (no is reference)	1.69 (0.36 to 8.01), *p *=* *0.506	a
Unstable housing		
Yes (no is reference)	1.36 (0.29 to 6.35), *p *=* *0.694	a
Full‐time female		
Yes (no is reference)	1.11 (0.24 to 5.07), *p *=* *0.889	a
Hormone use		
Yes (no is reference)	1.42 (0.32 to 6.36), *p *=* *0.645	a
Alcohol use in past 12 months		
Yes; >4 drinks in a sitting (no is reference)	0.97 (0.16 to 5.83), *p *=* *0.976	a
Substance use in past 12 months		
Yes (no is reference)	*2.43 (0.54 to 10.99), p = 0.245*	2.58 (0.72 to 9.23), *p *=* *0.143
Gender discrimination		
Yes (no is reference)	0.42 (0.08‐ 2.19), *p *=* *0.299	a
Race discrimination		
Yes (no is reference)	*2.72 (0.56 to 13.14), p = 0.211*	1.91 (0.32 to 11.33), *p *=* *0.474
Gender and race discrimination		
Yes (no is reference)	***6.28 (1.37 to 28.70), p = 0.018***	**5.14 (1.07 to 24.68), ** ***p *** **=** *** *** **0.041**

Italics: included in the multivariable model as overall *p *<* *0.25. Bold values: statistically significant (*p *<* *0.05). RDS, respondent‐driven sampling.

a Not included in model.

### 3.2 Correlates of not currently on ART

Thirteen per cent of the population of transwomen living with HIV were not on ART. In Table 4, the bivariate analysis is presented for those currently not on ART. Transwomen who experienced both anti‐trans discrimination and racism had 5.46 times higher odds of not being on ART than those who did not report both types of discriminations (95% CI: 1.38 to 21.53; *p *=* *0.016). Model‐building criteria informed a final multivariable model that included race, education, current hormone use, substance use, racism only and both anti‐trans discrimination and racism. In the multivariable logistic regression model, transwomen who currently used hormones had significantly lower adjusted odds of *not* being on ART than those not on hormones (aOR: 0.30; 95% CI: 0.09 to 0.92; *p *=* *0.036) (Table 4).

**Table 4:** Bivariate and multivariable analysis: RDS‐weighted estimates of transwomen living with HIV not on ART in San Francisco, 2016 to 2017 (n = 123)

Demographics	Not on ART Reference group = On ART	
	OR (95% CI), *p*	Adjusted odds ratio (95% CI), *p*
Age (23 to 71 years)		
20 to 29 years (reference)	‐	a
30 to 39 years	0.73 (0.12 to 4.37), *p *=* *0.729	
40 to 49 years	0.52 (0.09 to 2.95), *p *=* *0.461	
50 to 59 years	0.28 (0.04 to 1.78), *p *=* *0.174	
60 to 71 years	1.64 (0.17 to 15.68), *p *=* *0.665	
Gender identity		
Female (transgender women reference)	1.61 (0.53 to 4.94), *p *=* *0.398	a
Race/ethnicity		
Other (reference)	*‐*	‐
African American	*2.36 (0.53 to 10.45), p = 0.255*	2.06 (0.42 to 10.05), *p *=* *0.369
Latina	*0.90 (0.19 to 4.23), p = 0.894*	0.68 (0.12 to 4.00), *p *=* *0.666
Education		
Less than high school (reference)	*‐*	‐
High school diploma	*0.70 (0.20 to 2.49), p = 0.575*	0.37 (0.10 to 1.40), *p *=* *0.142
More than high school	*2.20 (0.60 to 8.12), p = 0.233*	2.26 (0.61 to 8.39), *p *=* *0.222
Born in the US		
Yes (no is reference)	1.26 (0.34 to 4.61), *p *=* *0.730	a
Unstable housing		
Yes (no is reference)	1.55 (0.46 to 5.19), *p *=* *0.475	a
Full‐time female		
Yes (no is reference)	1.76 (0.57 to 5.43), *p *=* *0.319	a
Hormone use		
Yes (no is reference)	*0.46 (0.15 to 1.41), p = 0.172*	**0.30 (0.09 to 0.92), ** ***p *** **=** *** *** **0.036**
Alcohol use in past 12 months		
Yes; >4 drinks in a sitting (no is reference)	1.87 (0.42 to 8.22), *p *=* *0.407	a
Substance in past 12 months		
Yes (no is reference)	*2.31 (0.74 to 7.27), p = 0.150*	1.96 (0.46 to 8.36), *p *=* *0.363
Gender discrimination		
Yes (no is reference)	1.31 (0.35 to 4.83), *p *=* *0.687	a
Race discrimination		
Yes (no is reference)	*2.21 (0.63 to 7.77), p = 0.212*	1.21 (0.26 to 5.66), *p *=* *0.808
Gender and race discrimination		
Yes (no is reference)	***5.46 (1.38 to 21.53), p = 0.016***	5.46 (0.93 to 32.12), *p *=* *0.060

Italics: included in the multivariable model as overall *p *<* *0.25. Bold values: statistically significant (*p *<* *0.05). ART, antiretroviral therapy; RDS, respondent‐driven sampling.

a Not included in model.

### 3.3 Correlates of detectable/unknown viral load

Of transwomen living with HIV, the population estimate of transwomen with a self‐reported detectable viral load was 22.2% and 13.5% with an unknown viral load (Table 5). The relative risk (RR) of 40 to 49 years old transwomen with unknown viral load was significantly lower by 0.06 than those in the 20 to 29 years old group (95% CI: 0.01 to 0.43; *p *=* *0.006). Those in the 50 to 59 years old age group with unknown viral load had a lower RR of 0.13 (95% CI: 0.02 to 0.97; *p *=* *0.046) than 20 to 29 years old group. Furthermore, those with unstable housing had a 6.42 higher RR of having a detectable viral load than those in stable housing (95% CI: 1.27 to 32.48; *p *=* *0.025). Age, housing instability and experiences of both anti‐trans discrimination and racism were included in the final model as the *p *<* *0.25. The adjusted RR (aRR) of a detectable viral load among participants with unstable housing was 7.37 times greater than those with stable housing (95% CI: 1.07 to 50.88; *p *=* *0.043). The aRR of an unknown viral load among the 40 to 49 years old transwomen compared to the 20 to 29 years old group was 0.06 times lower (95% CI: 0.01 to 0.52; *p *=* *0.011). No other associations were found.

**Table 5:** Bivariate and multivariable analysis: RDS‐weighted estimates of transwomen living with HIV with detectable and unknown viral load in San Francisco, 2016 to 2017 (n = 123)

Demographics	Detectable and unknown viral load Reference group = undetectable viral load			
	Detectable viral load	Unknown viral load	Detectable viral load	Unknown viral load
	RR (95% CI), *p*	RR (95% CI), *p*	ARR (95% CI), *p*	ARR (95% CI), *p*
Age (23 to 71 years)				
20 to 29 years (reference)	*‐*	*‐*		
30 to 39 years	*0.81 (0.08 to 8.65), p = 0.859*	*0.19 (0.02 to 1.62), p = 0.129*	1.12 (0.09 to 14.51), *p *=* *0.931	0.24 (0.03 to 2.25), *p *=* *0.210
40 to 49 years	*0.32 (0.03 to 3.33), p = 0.339*	***0.06 (0.01 to 0.43), p = 0.006***	0.58 (0.05 to 6.60), *p *=* *0.655	**0.06 (0.01 to 0.52), ** ***p *** **=** *** *** **0.011**
50 to 59 years	*1.01 (0.09 to 11.74), p = 0.993*	***0.13 (0.02 to 0.97), p = 0.046***	2.05 (0.16 to 26.64), *p *=* *0.580	0.13 (0.02 to 1.05), *p *=* *0.055
60 to 71 years	*0.78 (0.03 to 17.39), p = 0.874*	*0.54 (0.04 to 6.75), p = 0.629*	2.68 (0.13 to 54.58), *p *=* *0.518	0.79 (0.06 to 10.19), *p *=* *0.857
Gender identity				
Female (transgender women reference)	2.62 (0.58 to 11.78), *p *=* *0.208	1.48 (0.47 to 4.70), *p *=* *0.501	a	a
Race/ethnicity				
Other (reference)	‐	‐	a	a
African American	0.30 (0.06 to 1.49), *p *=* *0.138	0.99 (0.24 to 4.06), *p *=* *0.984		
Latina	0.61 (0.11 to 3.53), *p *=* *0.580	0.42 (0.11 to 1.59), *p *=* *0.199		
Education				
Less than high school (reference)	‐	‐	a	a
High school diploma	0.93 (0.15‐ 5.80), *p *=* *0.937	0.91 (0.23 to 3.53), *p *=* *0.891		
More than high school	0.95 (0.17‐ 5.27), *p *=* *0.951	1.52 (0.38 to 6.02), *p *=* *0.548		
Born in the US				
Yes (no is reference)	1.19 (0.15 to 9.19), *p *=* *0.870	0.83 (0.25 to 2.71), *p *=* *0.752	a	a
Unstable housing				
Yes (no is reference)	***6.42 (1.27 to 32.48), p = 0.025***	*1.20 (0.38 to 3.76), p = 0.757*	**7.37 (1.07 to 50.88), ** ***p *** **=** *** *** **0.043**	0.71 (0.20 to 2.53), *p *=* *0.595
Full‐time female				
Yes (no is reference)	2.99 (0.66 to 13.50), *p *=* *0.154	1.69 (0.53 to 5.40), *p *=* *0.371	a	a
Hormone use				
Yes (no is reference)	0.72 (0.15 to 3.50), *p *=* *0.680	0.46 (0.14 to 1.51), *p *=* *0.199	a	a
Alcohol use in past 12 months				
Yes; >4 drinks in a sitting (no is reference)	0.58 (0.06 to 5.64), *p *=* *0.637	1.58 (0.34 to 7.25), *p *=* *0.553	a	a
Substance use in past 12 months				
Yes (no is reference)	1.70 (0.35 to 8.34), *p *=* *0.509	1.19 (0.38 to 3.77), *p *=* *0.765	a	a
Gender discrimination				
Yes (no is reference)	1.11 (0.19 to 6.45), *p *=* *0.904	0.92 (0.25 to 3.46), *p *=* *0.906	a	a
Race discrimination				
Yes (no is reference)	0.50 (0.10 to 2.52), *p *=* *0.400	1.75 (0.49 to 6.26), *p *=* *0.385	a	a
Gender and race discrimination				
Yes (no is reference)	*4.57 (0.79 to 26.37), p = 0.088*	*3.44 (0.99 to 11.92), p = 0.052*	4.08 (0.70 to 23.95), *p *=* *0.118	3.36 (0.86 to 13.19), *p *=* *0.081

Italics: included in the multivariable model as overall *p *<* *0.25. Bold values: statistically significant (*p *<* *0.05). aRR, adjusted RR; RDS, respondent‐driven sampling; RR, relative risk ratio, probability of event occurring in the exposed group compared to occurrence in the non‐exposed group.

a Not included in model.

## 4 Discussion

We found high rates of HIV care access and ART use among transwomen living with HIV in San Francisco, although self‐reported viral load rates were lower. Approximately 86% accessed HIV care, 87% were on ART and 64% self‐reported an undetectable viral load. Our findings on HIV care engagement and treatment use were inconsistent with other studies that previously found low HIV care continuum indicators for transwomen relative to all those in San Francisco living with HIV 4, 5, 6, 7, 8, 9, 10. Previous data from 2014 in San Francisco found that 65% were on ART and only 44% reported viral suppression 10. This study suggests a significant improvement in HIV care and treatment access for transwomen in San Francisco in the past two years. Transwomen may have benefited from the increased push for HIV elimination in San Francisco that began in 2013. Partnerships were formed between community clinics, academia and public health organizations in San Francisco to push outreach and HIV testing programmes to reach the trans community 21. This extra push may have complimented work already being done in San Francisco's long‐standing trans health clinics and health department efforts for rapid linkage to HIV care for all San Franciscans 22.

Barriers and facilitators to optimal HIV care that can be a focus of policies and interventions to improve transwomen's HIV care outcomes were also identified. We found that age, current hormone use, unstable housing and the combined experience of anti‐trans discrimination and racism were significantly associated with poor HIV care continuum outcomes. Our study's result on age indicates that older transwomen in their 40s are less likely to not be in care and are at less risk of having an unknown viral load compared to transwomen in their 20s. These findings may be due to successful programmes that link HIV care among older transwomen. These results highlight an opportunity to increase efforts in HIV care among young transwomen.

Cross‐sex hormone therapy was associated with higher odds of ART use among transwomen living with HIV, while unstable housing and experiences of both anti‐trans discrimination and racism were barriers to ART use and viral suppression. Our results on the protective effect of hormones are contrary to prior studies that report transwomen prioritize hormone use over ART 6, 23, 24. Transwomen reported limited finances, transportation concerns and fear of discrimination resulting in choosing cross‐sex hormone therapy (which induces desired feminizing features among transwomen) over HIV care 23, 24, 25, 26. However, community clinics in San Francisco offer both trans healthcare and HIV care, which may reduce the need to prioritize one type of care over another 21, 27. Our contrary findings may support the notion that models of HIV care for transwomen will benefit from integration of trans‐specific care.

Our results support past studies showing that among all people living with HIV, unstable housing is associated with a detectable viral load 27, 28, 29. The high prevalence of transwomen with unstable housing in our study underscores the impact of housing on this population, and may, in part, be explained by reports of housing discrimination based on gender presentation 25, 26. The loss of social and familial support, which may be due to anti‐trans discrimination may also lead to a loss of stable housing 25, 26. A recent study conducted in San Francisco found that for people living with HIV, the degree of unstable housing (from single room occupancy, staying with friends, shelter or outdoors) was associated with lower odds of viral load suppression 29. Policies that promote access to stable housing for transwomen may mitigate high‐risk behaviours, and for those who are living with HIV, may be a facilitator to HIV care.

Previous studies found that discrimination had a significant impact on HIV care continuum indicators for transwomen living with HIV 6, 24, 25, 26, 28, 29, 30, 31, 32, 33. Prior experiences of anti‐trans discrimination were associated with delays in HIV testing among transwomen in Brazil and Jamaica 32, 33. The effects of anti‐trans discrimination may be particularly pronounced for transwomen of colour, who also face racism and bear the largest burden of HIV in the transwomen population 23, 24, 31.

Our study is unique in that it allows us to compare the effects of anti‐trans discrimination, racism and the combination of these two types of discrimination on HIV care continuum indicators among transwomen. We found that when measured independently, racism and anti‐trans discrimination were not associated with poor health outcomes, but when combined, discrimination was associated with not accessing HIV care. This finding supports the intersectional framework, which suggests that race and gender identity occur simultaneously and are not independent of each other and should be measured together to capture the intersectional effects of these identities 31, 34, 35.

This study has limitations. The study's cross‐sectional design limited the study due to the conditional outcomes (on ART to have suppressed viral load), a Markov‐based analysis may lead to a better interpretation of data. Given the wide confidence intervals for housing and multiple discriminations, the results indicate that these variables need more research with larger numbers of participants to determine significance. In addition, some important associations may be missed as the sample size was not large enough to detect significance. It is important to note that relative to the size of the transwomen population in San Francisco, this is a sizeable sample. A conservative estimate of the size of the transwomen population in San Francisco is 1000 transwomen 36. Thus, this study sample may make up 10% population of transwomen at risk of HIV in San Francisco, speaking to the importance of this study for understanding the HIV care continuum in this population.

Furthermore, this present study was limited in the ability to analyse Native Hawaiian, Pacific Islander, Asian, White and multiple races separately, highlighting the need for future studies to focus on these communities. Additionally, the use of SS weights and transferring RDS data from RDS Analyst to STATA does not necessarily ensure accurate inference to the population at large potentially limiting the ability to control for bias 37. The study oversampled transwomen with lower SES in San Francisco and cannot be generalizable to all San Francisco transwomen or to transwomen living in other geographical areas. Furthermore, San Francisco's policies and resources may differ from other cities thereby limiting the generalizability of the results. Self‐reported findings, particularly in the EOD scale and the self‐reported viral load may be influenced by social desirability or recall bias and could inflate estimates and compromise the accuracy of findings. Given the design of the study, the self‐reported viral load could not be verified. Despite these limitations, this analysis is the first of its kind to look at HIV care continuum outcomes with structural indicators and can inform future longitudinal studies with larger samples.

## 5 Conclusions

Despite important barriers to health for transwomen in San Francisco, this city's response to HIV may provide a model for HIV treatment access and elimination in this important key population. Our analysis was restricted to within differences of transwomen living with HIV and their uptake of care, next steps for research will provide insight of the uptake of HIV prevention services between HIV‐negative transwomen and transwomen living with HIV. Studies to elucidate how transwomen's health needs are being addressed in San Francisco are needed. Also, efforts to improve viral load suppression are sorely needed as one‐fifth in our sample were not virally suppressed despite 100% treatment access in San Francisco. Interventions to increase viral suppression will need to address the co‐occurring effect of anti‐trans discrimination and racism inside and outside the healthcare system, including housing availability and discrimination. Without efforts to address structural barriers outside clinic walls, there may be little hope of HIV elimination in San Francisco and other cities around the world.

## Competing interests

None.

## Authors’ contributions

GNB conceptualized the paper and ran data analysis. HFR designed and implemented the original study and contributed to the intellectual content. GNB, CT and ECW drafted the manuscript and edited the drafts. JL, GMS and CDR contributed to the intellectual content. All authors reviewed and approved the manuscript for submission.

## References

[1] Center for Disease Control . HIV incidence: estimate annual infections in the U.S., 2008‐2014. 2017 [cited 2017 Jul 1]. Available at: https://www.cdc.gov/nchhstp/newsroom/docs/factsheets/hiv-incidence-fact-sheet_508.pdf

[2] Baral SD , Poteat T , Strömdahl S , Wirtz AL , Guadamuz TE , Beyrer C . Worldwide burden of HIV in transgender women: a systematic review and meta‐analysis. Lancet Infect Dis. 2013;13(3):214–22. 23260128 10.1016/S1473-3099(12)70315-8

[3] Joint United Nations Programme on HIV/AIDS . 90‐90‐90 an ambitious treatment target to help end the AIDS epidemic. 2014[cited 2017 Jul 27]. Available at: http://www.unaids.org/sites/default/files/media_asset/90-90-90_en.pdf

[4] Mizuno Y , Frazier EL , Huang P , Skarbinski J . Characteristics of transgender women living with HIV receiving medical care in the United States. LGBT Health. 2015;2(3):228–34. 26788671 10.1089/lgbt.2014.0099 PMC6711156

[5] Poteat T , Scheim A , Xavier J , Resiner S , Baral S . Global epidemiology of HIV infection and related syndemics affecting transgender people. J Acquir Immune Defic Syndr. 2016;72(3):s210–9. 27429185 10.1097/QAI.0000000000001087 PMC4969059

[6] Sevelius JM , Saberi P , Johnson MO . Correlates of antiretroviral adherence and viral load among transgender women living with HIV. AIDS Care. 2014;26(8):976–82. 24646419 10.1080/09540121.2014.896451 PMC4054817

[7] Jalil EM , Wilson EC , Luz PM , Velasque L , Moreira RI , Castro CV , et al. HIV testing and the care continuum among transgender women: population estimates from Rio de Janeiro, Brazil. J Int AIDS Soc. 2017;20:21873. 28953323 10.7448/IAS.20.1.21873 PMC5640309

[8] Reisner SL , Jadwin‐Cakmak L , White Hughto JM , Martinez M , Salomon L , Harper GW . Characterizing the HIV prevention and care continua in a sample of transgender youth in the U.S. AIDS Behav. 2017; 21(12):3312–27. 29138982 10.1007/s10461-017-1938-8 PMC5705332

[9] Bukowski LA , Chandler CJ , Creasy SL , Matthews DD , Friedman MR , Stall RD . Characterizing the HIV care continuum and identifying barriers and facilitators to HIV diagnosis and viral suppression among Black transgender women in the United States. J Acquir Immune Defic Syndr. 2018;79(4):413–20. 30080750 10.1097/QAI.0000000000001831 PMC6521857

[10] Santos G , Wilson EC , Rapues J , Macias O , Packer T , Raymond HF . HIV treatment cascade among transgender women in San Francisco respondent driven sampling study. Sex Transm Infect. 2014;90(5):430–3. 24714446 10.1136/sextrans-2013-051342

[11] Scheer S , Hsu L , Schwarcz S , Pipkin S , Havlir D , Buchbinder S , et al. Trends in the San Francisco human immunodeficiency virus epidemic in the “Getting to Zero” Era. Clin Infect Dis. 2018;66(7):1027–34. 29099913 10.1093/cid/cix940 PMC6248750

[12] Rapues J , Wilson EC , Packer T , Colfax GN , Raymond HF . Correlates of HIV infection among transfemales, San Francisco, 2010: results from a respondent‐driven sampling study. Am J Public Health. 2013;103(8):1485–92. 23763398 10.2105/AJPH.2012.301109 PMC4007863

[13] National Institute on Alcohol Abuse and Alcoholism . Rethinking drinking alcohol and your health. NIH No. 15‐3770. 2010 [cited Accessed 2017 Jun 01]. Available at: https://pubs.niaaa.nih.gov/OrderForm/EncForm/order_page

[14] Krieger N , Smith K , Naishadham D , Hartman C , Barbeau EM . Experiences of discrimination: validity and reliability of a self‐report measure for population health research on racism and health. Soc Sci Med. 2005;61(7):1576–96. 16005789 10.1016/j.socscimed.2005.03.006

[15] Van Dyke ME , Vaccarino V , Dunbar SB , et al. Socioeconomic status discrimination and C‐reactive protein in African‐American and white adults. Psychoneuroendocrinology. 2017;82:9–16. 28482209 10.1016/j.psyneuen.2017.04.009 PMC5519320

[16] Handcock MS , Fellows IE , Gile K . RDS Analyst: software for the analysis of respondent‐driven sampling, Version 0.42. 2014. [cited Accessed 2017 Jun 20]. Available at: http://hpmrg.org

[17] Gile KJ . Improved inference for respondent‐driven sampling data with application to HIV prevalence estimation. J Am Stat Assoc. 2011;106(493):135–46.

[18] HIV epidemiology section . HIV/AIDS epidemiology annual report 2010. San Francisco, CA: San Francisco Department of Public Health AIDS Office; 2011.

[19] Raymond HF , Wilson EC , McFarland W . Transwomen population size [letter]. Am J Public Health. 2017;107(9):e12. 10.2105/AJPH.2017.303964 PMC5551612 28787216

[20] Bursac Z , Gauss CH , Williams DK , Hosmer DW . Purposeful selection of variables in logistic regression. Source Code Biol Med. 2008;3:17. 19087314 10.1186/1751-0473-3-17 PMC2633005

[21] Rebchook G , Keatley J , Contreras R , Perloff J , Molano LF , Reback CJ , et al. The Transgender Women of Color Initiative: implementing and evaluating innovative interventions to enhance engagement and retention in HIV care. Am J Public Health. 2017;107:224–9. 28075641 10.2105/AJPH.2016.303582 PMC5227951

[22] Reisner SL , Radix A , Deutsch MB . Integrated and gender affirming transgender clinical care and research. J Acquir Immune Defic Syndr. 2016;72 Suppl 3:S235–42. 27429189 10.1097/QAI.0000000000001088 PMC4969060

[23] Melendez RM , Pinto RM . HIV prevention and primary care for transgender women in community‐based clinic. J Assoc Nurses AIDS Care. 2009;20(5):387–97. 19732697 10.1016/j.jana.2009.06.002 PMC3534725

[24] Sevelius JM , Patouhas E , Keatley JG , Johnson MO . Barriers and facilitators to engagement and retention in care among transgender women living with human immunodeficiency virus. Ann Behav Med. 2014;47(1):5–16. 24317955 10.1007/s12160-013-9565-8 PMC3925767

[25] Grant JM , Mottet LA , Tanis J , Harrison J , Herman JL , Keisling M . Injustice at every turn: a report of the national transgender discrimination survey. Washington, DC: National Center for Transgender Equality and National Gay and Lesbian Task Force; 2011.

[26] Wilson EC , Arayasirikul S , Johnson K . Access to HIV care and support services for African American transwomen living with HIV. Int J Transgend. 2013;14(4):182–95. 24817835 10.1080/15532739.2014.890090 PMC4012687

[27] Wilson EC , Turner C , Arayasirikul S , Woods T , Nguyen T , Lin R , et al. Housing and income effects on HIV‐related health outcomes in the San Francisco Bay Area‐ findings from the SPNS transwomen of color initiative. AIDS Care. 2018;30:1356–9. 29920118 10.1080/09540121.2018.1489102

[28] Aidala AA , Wilson MG , Shubert V , Gogolishvili D , Globeman J , Rueda S , et al. Housing status, medical care, and health outcomes among people living with HIV/AIDS: a systematic review. Am J Public Health. 2016;106(1):e1–22. 10.2105/AJPH.2015.302905 PMC4695926 26562123

[29] Clemenzi‐Allen A , Geng E , Christopoulos K , Hammer H , Buchbinder S , Havlir D , et al. Degree of housing instability shows independent “dose‐response” with virologic suppression rates among people living with human immunodeficiency virus. Open Forum Infect Dis. 2018; 5(3):ofy035. 29577059 10.1093/ofid/ofy035 PMC5850870

[30] Remien RH , Bauman LJ , Mantell JE , Tsoi B , Lopez‐Rios J , Chhabra R , et al. Barriers and facilitators to engagement of vulnerable populations in HIV primary care in New York City. J Acquir Immune Defic Syndr. 2015;69 Suppl 1:s16–24. 25867774 10.1097/QAI.0000000000000577 PMC4559146

[31] Logie CH , James L , Tharao W , Loutfy MR . HIV, gender, race, sexual orientation, and sex work: a qualitative study of intersectional stigma experienced by HIV‐positive women in Ontario, Canada. PLoS Med. 2011;8(11):e1001124. 22131907 10.1371/journal.pmed.1001124 PMC3222645

[32] Logie CH , Wang Y , Lacombe‐Duncan A , Jones N , Ahmed U , Levermore K , et al. Factors associated with sex work involvement among transgender women in Jamaica: a cross‐sectional study. J Int AIDS Soc. 2017;20(1):21422. 28406598 10.7448/IAS.20.01/21422 PMC5515035

[33] Pinheiro Junior FM , Kendall C , Martins TA , Mota RMS , Macena RHM , Glick J , et al. Risk factors associated with resistance to HIV testing among transwomen in Brazil. AIDS Care. 2016;28(1):92–7. 10.1080/09540121.2015.1066751 26274065

[34] Crenshaw K . Mapping the margins: intersectionality, identity politics, and violence against women of color. Stanford Law Rev. 1991;43(6):1241–99.

[35] Bowleg L . The problem with the phrase women and minorities: intersectionality‐an important theoretical framework for public health. Am J Public Health. 2012;102(7):1267–73. 22594719 10.2105/AJPH.2012.300750 PMC3477987

[36] Wesson P , Qabazard RF , Wilson EC , McFarland W , Raymond HF . Estimating the population size of transgender women in San Francisco using multiple methods, 2013. Int J Transgend. 2017;19(1):107–12.

[37] World Health Organization . Introduction to HIV/AIDS and sexually transmitted infection surveillance module 4: supplement a guide to using RDS analyst and NetDraw. 2014.

